# Miscellaneous Medical Intelligence

**Published:** 1849-09

**Authors:** 


### ARTICLE III.

### MISCELLANEOUS INTELLIGENCE.

G. N. Fitch, M. D., late Professor of Principles and Practice of Medicine in Rush Medical College, has been elected to Congress from the Northern District in Indiana. Aside from giving any expression of a political partizan character, we believe he will be an able and efficient representative. We hope he may, by efforts in procuring a law against patenting nostrums, even more highly distinguish himself, than has Dr. Edwards of Ohio, by the Drug Inspection Law. It is high time the National Government had withdrawn its patronage from the quackery of patenting medical compounds and systems of practice.

American fondness for any thing that comes through European channels is strongly illustrated by an extract from a Paris Journal translated by Dr. D. W. Yandell, reporting the cure of Spina Bifida by iodine injections by Dr. Brainard, and credited to an Indiana Journal, which a number of our exchanges are copying The case was reported by Prof. Brainard in the January 1848 number of this Journal, then under the title of the Illinois and Indiana Medical and Surgical Journal, but passed unnoticed by most of our cotemporaries. But now that it has been translated into French and back again into English, having suffered some in point of correctness, it seems to attract considerable attention.

Professor Davis arrived in Chicago, the field of his future labors, a few days since, where he has taken up his permanent residence. The Annalist, in consequence of his leaving New York, has been discontinued.

Dr. E. D. Fenner, of New Orleans, proposes to publish an annual volume to be entitled *Southern Medical Reports*, which, from his ability and the facilities he will enjoy for collecting the information he proposes to embody in them—the Meterology, Topography and Diseases of the Southern States—will be a valuable work.

